# Cyclin-Dependent Kinase Inhibitor Gene *TaICK1* acts as a Potential Contributor to Wheat Male Sterility induced by a Chemical Hybridizing Agent

**DOI:** 10.3390/ijms21072468

**Published:** 2020-04-02

**Authors:** Lili Zhang, Chaojie Wang, Yongang Yu, Yamin Zhang, Yulong Song, Zheng Li, Shuping Wang, Yanfang Zhang, Xiaofeng Guo, Dan Liu, Ziliang Li, Shoucai Ma, Jinjuan Zheng, Huiyan Zhao, Gaisheng Zhang

**Affiliations:** 1 College of Agronomy, Northwest A & F University, National Yangling Agricultural Biotechnology & Breeding Center, Yangling 712100, Shaanxi, China; zll3142015@163.com (L.Z.); superjie_wang@nwafu.edu.cn (C.W.); ymzhang2017@163.com (Y.Z.); Sylbl1986@aliyun.com (Y.S.); Sylbl1986@aliyun.com (Z.L.); 18392434431@163.com (X.G.); 15754507101@163.com (D.L.); 15721911215@163.com (Z.L.); mashoucai@sohu.com (S.M.);; 2 Yangling Branch of State Wheat Improvement Centre, Yangling 712100, Shaanxi, China; 3 Wheat Breeding Engineering Research Center, Ministry of Education, Yangling 712100, Shaanxi, China; 4 Key Laboratory of Crop Heterosis of Shaanxi Province, Yangling 712100, Shaanxi, China; 5 Department of Life Sciences, Henan Institute of Science and Technology, Xinxiang 453000, Henan, China; xkyya@163.com; 6 Hubei Key Laboratory of Waterlogging Disaster and Agricultural Use of Wetland, College of Agronomy, Yangtze University, Jingzhou 434000, Hubei, China; wangshuping2003@126.com; 7 College of Plant Protection, Northwest A & F University, Yangling 712100, Shaanxi, China; zhaohy@nwsuaf.edu.cn

**Keywords:** wheat, male sterility, CHA SQ-1, TaICK1, seed setting rate

## Abstract

Heterosis has been widely accepted as an effective strategy to increase yields in plant breeding. Notably, the chemical hybridization agent SQ-1 induces male sterility in wheat, representing a critical potential tool in hybrid seed production. However, the mechanisms underlying the male sterility induced by SQ-1 still remain poorly understood. In this study, a cyclin-dependent kinase inhibitor gene, *TaICK1*, which encodes a 229 amino acid protein, was identified as a potential contributor to male sterility in common wheat. The expression of *TaICK1* was upregulated during the development of anthers in Xinong1376 wheat treated with SQ-1. Meanwhile, the seed setting rate was found to be significantly decreased in *TaICK1* transgenic rice. Furthermore, we identified two cyclin proteins, *TaCYCD2;1* and *TaCYCD6;1*, as interactors through yeast two-hybrid screening using TaICK1 as the bait, which were validated using bimolecular fluorescence complementation. Subcellular localization revealed that the proteins encoded by *TaICK1*, *TaCYCD2;1,* and *TaCYCD6;1* were localized in the cell nucleus. The expression levels of *TaCYCD2;1* and *TaCYCD6;1* were lower in Xinong1376 treated with SQ-1. A further analysis demonstrated that the expression levels of *OsCYCD2;1* and *OsCYCD6;1* were lower in transgenic *TaICK1* rice lines as well. Taken together, these results suggest that the upregulation of *TaICK1,* induced by SQ-1, may subsequently suppress the expression of *TaCYCD2;1* and *TaCYCD6;1* in anthers, resulting in male sterility. This study provides new insights into the understanding of SQ-1-induced wheat male sterility, as well as the developmental mechanisms of anthers.

## 1. Introduction

Bread wheat (***Triticum aestivum L.***) is one of the most stable cereal crops and feeds nearly 40% of the world population, contributing to approximately 20% of the global total caloric intake [1,2,3]. In China, wheat is the third most important food crop after rice and maize, in terms of either total production or sown area. In 2017, the area of sown wheat was more than 24.5 million hectares, with a total output of 1.34 hundred million tons (http://www.stats.gov.cn/tjsj/ndsj/2018/indexch.htm, China Statistical Yearbook 2018). However, with the continued increase in the global population, reductions in cultivatable land (19% less in the wheat cultivated area in 2016 compared to 1998), frequent abnormal climate changes, and other factors, a gap still remains between the total output of wheat and its demand [4,5]. Therefore, there is still an urgent need to improve wheat yields [3,6].

Heterosis is one of the most effective ways to improve the yield and quality of wheat [7,8]. At present, two main wheat heterosis strategies are available: a genetic male sterility system (e.g., CMS, cytoplasmic male sterility; or PTMS, photo-thermo-sensitive male sterility) and a physiological male sterility (PHYMS) system (e.g., CIMS, chemically induced male sterility) [9]. For the genetic male sterile system, the creation of excellent male-sterile lines, maintainer lines, and restorer lines remains a roadblock for the production of hybrid seed [10]. By contrast, hybrid seed production using the CIMS system does not have these limitations [11].

Previous studies have indicated that the chemical hybridization agent (CHA) SQ-1 is an effective male-killing agent for wheat. It is worth noting that there are no evident interaction effects between most genotype varieties and SQ-1, nor negative effects on agronomic traits [12,13,14]. These results demonstrate that SQ-1 has tremendous application potential in wheat hybrid seed production. However, the degree of male sterility induced by SQ-1 in some fine varieties is unsatisfactory for hybrid seed production [15,16]. As a result, some fine varieties cannot be used as parents in hybrid seed production. Elucidation of the molecular mechanism underlying SQ-1-induced male sterility has the potential to improve the prospects for utilizing CHA. Nevertheless, the molecular mechanism underlying SQ-1-induced wheat male sterility remains relatively unknown, representing an impediment to the application of SQ-1-induced wheat male sterility. Previous research has indicated that pollen abortion may be associated with impaired energy metabolism after spraying of SQ-1 [17,18,19,20,21,22,23]. For example, SQ-1-induced sterility lines showed lower expression levels of the *PDH-E1α* gene, which is essential for regulation of the tricarboxylic acid (TAC) cycle [24]. This is consistent with a study showing that the inhibition of *PDH-E1α* expression in tobacco leads to male sterility [25]. In addition, pyruvate dehydrogenase kinase (PDK) is a core regulator of the cell cycle and can block the function of protein PDH-E1α through phosphorylation [26,27]. Meanwhile, *TaPCNA* is also an important regulator of *PDK* in wheat [28].

In our previous study, the protein TaICK1 was identified in a yeast two-hybrid assay using TaPCNA as the bait [29]. In plants, ICKs [30] are inhibitors of CDKs (cyclin-dependent kinases), which play a central role in regulating the cell cycle in plants [31]. To date, seven ICK proteins have been identified in the model plant *Arabidopsis thaliana*: ICK1/KRP1, ICK2/KRP2, KRP3, KRP4, KRP5, KRP6, and KRP7 [32]. Simultaneously, in other plants, increasingly more ICKs have also been identified, such as *NtKIS1a* and *NtKIS1b* in tobacco [33], *Zeama;KRP;1* and *Zeama;KRP;2* in maize [34], *LeKRP1* and *LeKRP2* in tomato [35], and *Orysa;KRP1* in rice [36].

It was reported that ICKs play an important role in the regulation of plant growth and development. ICKs can bind CDK–Cycd protein complexes directly to regulate the activity of CDKs [37] and, thus, regulate cell development [38]. Hence, a high level of ICKs can inhibit the CDK activity. For example, the overexpression of *NtKIS1a*, an ICK-like inhibitor isolated from tobacco, inhibits nuclear re-replication and reduces CDK activity in *Arabidopsis thaliana,* which demonstrates that plant organ size and shape as well as organ cell number and cell size might be controlled by the modulation of the activity of a single *NtKIS1a* gene [39]. Wang et al. (2000) reported that the growth of transgenic *35S::ICK1 Arabidopsis* plants is significantly inhibited, and most of their organs are smaller [40]. Similar results have been reported in other studies [39,40,41,42,43]. On the other hand, downregulation of both ICK4/KRP6 and ICK5/KRP7 can affect pollen development [32,44]. Furthermore, some members of the ICK protein family can interact with D-type cyclins (Cycds), which involves a number of CDK–Cycd protein complexes and plays a crucial role in the cell cycle [45]. For example, excess ICK2 can interact with CYCD2;1, thereby inhibiting lateral root formation [46]. Additionally, increased expression of *CYCD* can suppress the negative effects of excess ICK/KRP levels on plant growth [39,47]. These results suggest that ICKs may play an important role in the development of anther. Although there are some studies on the functions of ICK1 in anther development, relatively little is known about this process in common wheat. As wheat is one of the most important plants for humans, basic knowledge regarding the molecular biology of anther development will promote a better understanding of male sterility and, moreover, promote the utilization of heterosis.

In this study, we confirmed the full-length coding sequence of *TaICK1* in Xinong1376 (XN1376) wheat. The results show that *TaICK1* mRNA could be detected in roots, stems, leaves, developing seeds, and anthers. Remarkably, the transcript was most abundant in anthers, especially at the late uninucleate stage of anthers. Similar expression patterns of *TaICK1* were detected in the male sterile lines Xinong1376-CIMS (XN1376-CIMS), but the detected expression level was higher than that in XN1376 for all stages of anther development except at stage Bn. Meanwhile, the overexpression of *TaICK1* in rice may block the expression of cyclin (e.g., *OsCYCD6;1* and *OsCYCD2;1*). In addition, the seed setting rate significantly decreased in overexpressed *TaICK1* transgenic rice. This study provides a theoretical basis for further exploring the regulatory pathways related to energy metabolism in the process of SQ-1-induced male sterility in wheat and lays a foundation for eventually revealing the mechanism underlying wheat physiological male sterility induced by SQ-1.

## 2. Results

### 2.1. Morphological and Cytological Characteristics

**Figure 1:**
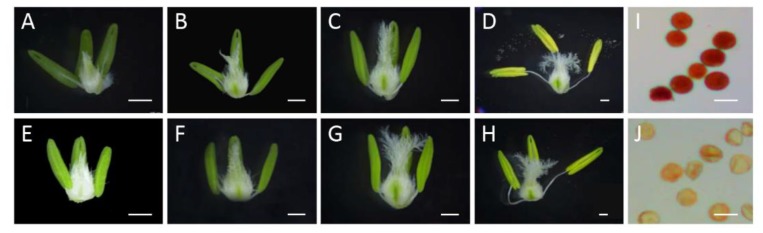
Anther phenotype and KI–I_2_ staining in XN1376 (**A**–**D**,**I**) and XN1376-CIMS (**E**–**H**,**J**). (**A**,**E**) Eun, early uninucleate stage; (**B**,**F**) Lun, late uninucleate stage; (**C**,**G**) Bn, binucleate stage; (**D**,**H**) Tn, trinucleate stage; and (**I**,**J**) KI–I_2_ staining. Scale bars are 1 mm in (**A**–**H**) and 50 µm in (**I**,**J**).

In this study, the development of wheat anthers was divided into four stages (e.g., Eun, the early uninucleate stage; Lun, the late uninucleate stage; Bn, the binucleate stage; and Tn, the trinucleate stage). As shown in Figure 1, the pistil tissue showed the normal development of all four stages of XN1376 and XN1376-CIMS. By contrast, the anthers from XN1376-CIMS showed a significant difference compared to XN1376 from the Eun stage to the Tn stage. For example, the anthers from XN1376-CIMS plants were smaller and lighter-colored than those from XN1376 plants (Figure 1A–H). More importantly, the anthers of XN1376-CIMS were shriveled and indehiscent, and less pollen could be deeply stained using the 1% KI–I_2_ solution compared to the anthers of XN1376 (Figure 1I,J). These results indicate that CHA SQ-1 had a negative effect on anther development and destroyed the activity of pollen grains.

**Figure 2:**
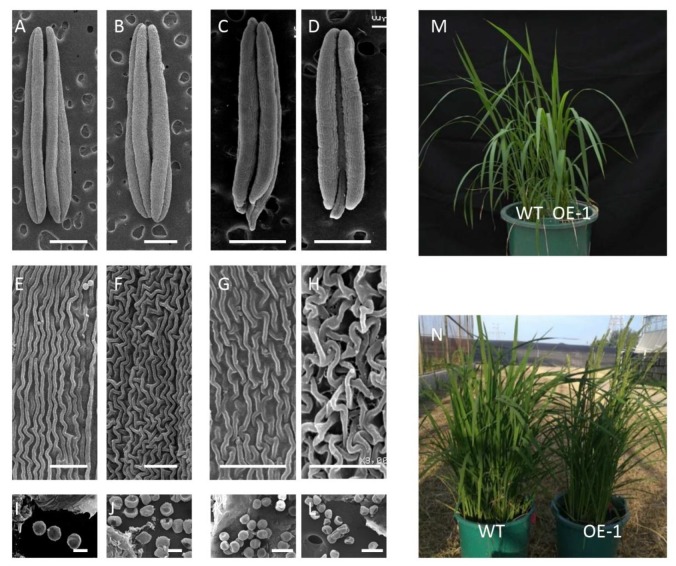
SEM observations of the mature pollen and phenotypes of transgenic rice plants. (**A**) Anthers of XN1376. (**B**) Anthers of XN1376-CIMS. (**C**) Anthers of wild-type Nipponbare. (**D**) Anthers of transgenic TaICK1 rice plants. (**E**) The outer epidermal cells of XN1376. (**F**) The outer epidermal cells of XN1376-CIMS. (**G**) The outer epidermal cells of wild-type Nipponbare. (**H**) The outer epidermal cells of transgenic TaICK1 rice plants. (**I**) The microspores of XN1376. (**J**) The microspores of XN1376-CIMS. (**K**) The microspores of wild-type Nipponbare. (**L**) The microspores of transgenic *TaICK1* rice plants. (**M**) The phenotype of *35S::TaICK1* overexpression plants in young seedlings compared to the wild type. The left is the wild type, and the right is the transgenic plant. (**N**) The phenotype of *TaICK1* overexpression plants in the heading period compared to the wild type. The left is the wild type and the right is the transgenic plant. Scale bars are 500 μm in **A**–**D**; 10 μm in **E**–**H**; and 50 μm in **I**–**L**.

The surface characteristics of the anthers and pollen grains at the Tn stage in XN1376-CIMS and XN1376 were analyzed using scanning electron microscopy (SEM) (Figure 2A,B,E,F,I,J). The anther surface characteristics were more irregular in shape in XN1376-CIMS compared to those in XN1376 (Figure 2E,F). In addition, the pollen grains exhibited a severely malformed, shrunken extine pattern, with small and shrunken germination apertures in XN1376-CIMS (Figure 2J). By contrast, the pollen grains from XN1376 looked round and did not shrink (Figure 2I).

### 2.2. Structure of the Gene *TaICK1*

**Figure 3:**
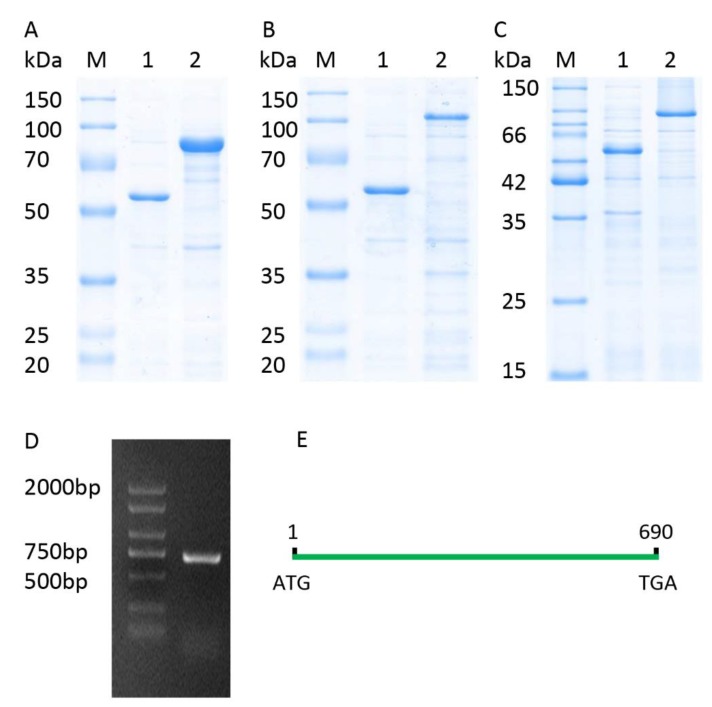
Recombinant expression analysis and full-length CDS of *TaICK1.* (**A**) Lane M: protein marker; lane 1: pCold-TF; lane 2: TaICK1 recombinant protein. (**B**) Lane M: protein marker; lane 1: pCold-TF; lane 2: TaCYCD2;1 recombinant protein. (**C**) Lane M: protein marker; lane 1: pCold-TF; lane 2: TaCYCD6;1 recombinant protein. (**D**) The full-length CDS of *TaICK1* was cloned by the reverse transcription polymerase chain reaction of the RNA from wheat XN1376 anthers. M: marker; 1: PCR product of *TaICK1*. (**E**) TaICK1 gene structure.

**Figure 4:**
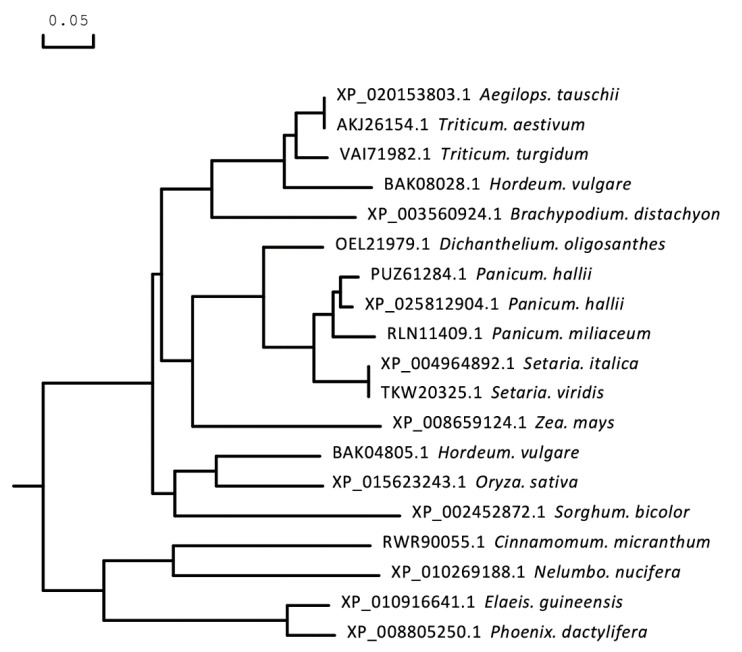
Phylogenetic tree based on the amino acid sequence of the protein TaICK1. A molecular phylogenetic analysis of the protein sequences of TaICK1 using the maximum likelihood method with bootstrap confidence values exceeding 80% from 1000 replicates. The number before the Latin name is the accession number from NCBI.

The full-length CDS of *TaICK1* was identified from XN1376 using PCR. The results of agarose electrophoresis show the presence of only one DNA band corresponding to a specific product (Figure 3D). Sequencing revealed that the gene *TaICK1* contained a 690 bp (Figure 3E) open reading frame that encoded 229 amino acid residues. The homology analysis indicated that the TaICK1 amino acid residue sequence has a high similarity with that of *Aegilops tauschii* (GenBank XP_020153803.1) and *Hordeum* (GenBank BAK08028.1)—99.56% and 83.26%, respectively (Figure 4).

### 2.3. Expression Level of *TaICK1* in XN1376-CIMS and XN1376

**Figure 5:**
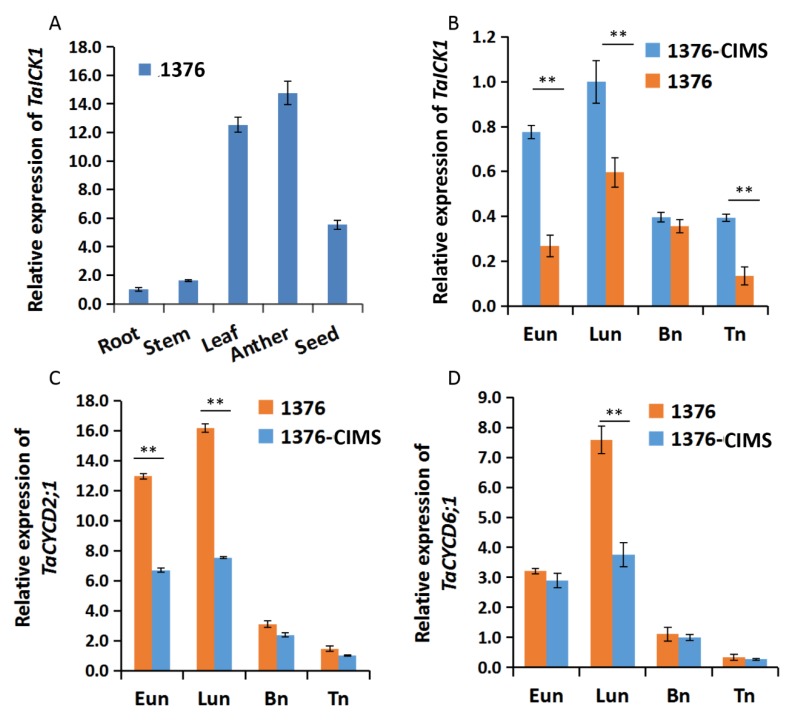
The expression patterns of *TaICK1*. (**A**) The expression pattern in various tissues of XN1376. (**B**) The expression levels of *TaICK1* in XN1376-CIMS and XN1376 at different stages of anther development. (**C**) The expression levels of *TaCYCD2;1* in XN1376-CIMS and XN1376 at different stages of anther development. (**D**) The expression levels of *TaCYCD6;1* in XN1376-CIMS and XN1376 at different stages of anther development. Data are presented as the means ± SD; mean and error bars indicate SD with three technical replicates and three independent biological replicates. The significance of differences was assessed using Student’s t-test. * *p* < 0.05, ** *p* < 0.01.

The expression patterns of *TaICK1* were first studied in various tissues of XN1376. Meanwhile, the expression patterns were further measured during the stages of Eun, Lun, Bn, and Tn in XN1376-CIMS and XN1376. The expression of *TaICK1* significantly differed depending on the tissue. As shown in Figure 5A, the most abundant cDNA of *TaICK1* was detected in anthers, followed by leaves and developing seeds. The root and stem presented less *TaICK1* cDNA compared to other tissues. Especially in root, the expression level was lower than 14 times that in anthers (Figure 5A). These results indicate that the expression pattern in wheat is organ-specific. In addition, the relative expression of *TaICK1* was further analyzed in the anthers of XN1376-CIMS and XN1376 and showed an increase from the Eun to Lun stage followed by a continuous decrease from the Lun to Tn stage in XN1376-CIMS. XN1376 also exhibited a similar expression pattern: the highest expression level was detected at the Lun stage (Figure 5B). Notably, the expression of *TaICK1* was higher in XN1376-CIMS than that in XN1376 at stages Eun, Lun, and Tn (Figure 5B).

### 2.4. The Phenotype of *TaICK1* Transgenic Rice Plants

The full-length CDS sequence of *TaICK1* was cloned into the expression vector pCAMBIA1301, which drives the expression of inserts through the 35S promoter. The vector was then introduced into the rice variety Nipponbare. In total, 15 independent *TaICK1* transgenic T_0_ plants were identified. Homozygous transgenic plants were obtained from the T_2_ lines. In young seedlings, there were no observable differences between the wild-type Nipponbare and transgenic plants (Figure 2M). By contrast, at later stages, one striking change was observed: the heading period was delayed by 7–10 days in transgenic plants compared to control plants (Figure 2N). In addition, the seed setting rates of transgenic plants and wild-type plants were investigated. The average seed setting rate was 46.5% in the T_1_ generation, which was significantly lower than that in the wild type (Table 1). To confirm the results, the homozygote T_2_ generation transgenic plants were selected for further analysis. Similar to the T_1_ generation, the average seed setting rate of the T_2_ generation was 50.2%, which was also significantly lower than that of the wild-type plants (Table 1).

SEM was used to observe the outside epidermal cells and pollen grains from transgenic and wild-type plants. The results showed that the anthers from the wild type were large and full (Figure 2C). By contrast, the anthers from the transgenic T_2_ generation were short and thin (Figure 2D). Meanwhile, the outside epidermal cells showed a roughly uniform distribution in wild type (Figure 2G), which was abnormal with relatively irregular distribution in the transgenic lines (Figure 2H). Further observations showed that the pollen grains of the wild type were plump, round, and spherical (Figure 2K), while the pollen grains of the transgenic plants were sunken into other irregular shapes (Figure 2L).

### 2.5. Expression Patterns of *TaICK1* and *OsICK1* in Transgenic Rice

**Figure 6:**
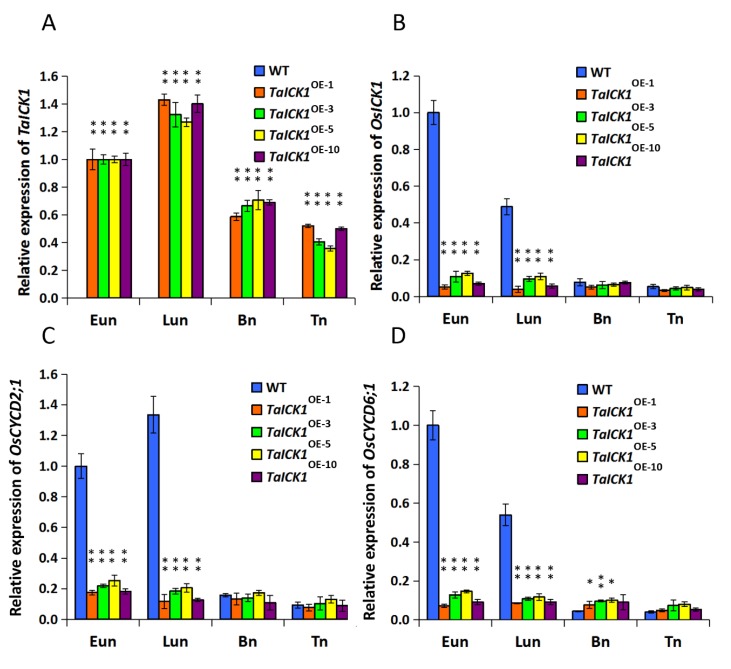
The expression levels of the genes *TaICK1*, *OsICK1*, *OsCYCD2;1*, and *OsCYCD6;1* in *TaICK1* transgenic rice and wild-type plants. (**A**) The expression levels of *TaICK1*. (**B**) The expression levels of *OsICK1*. (**C**) The expression levels of *OsCYCD2;1*. (**D**) The expression levels of *OsCYCD6;1*. Data are presented as the mean ± SD; the mean and error bars indicate SD from OE-1, OE-3, OE-5, and OE-10 with three independent biological and three technical replicates. The mRNA of *TaICK1* was not detected in the anthers of wild-type plants; OE: overexpression plants of T_2_; the number following OE is a code referring to an independent T_2_ overexpression plant. The significant differences were assessed using Student’s t-test. * *p* < 0.05, ** *p* < 0.01.

The expression patterns of *TaICK1* and *OsICK1* were examined in transgenic and wild-type rice plant anthers using qRT-PCR analyses (Figure 6A,B). The expression pattern of the *OsICK1* gene revealed that *OsICK1* had relatively higher expression levels in wild-type plants than those in transgenic plants during the Eun and Lun stages (Figure 6B). As shown in Figure 6A, the cDNA of *TaICK1* was detected only in the anthers of *TaICK1* transgenic plants.

### 2.6. Subcellular Localization Assay of Protein TaICK1

**Figure 7:**
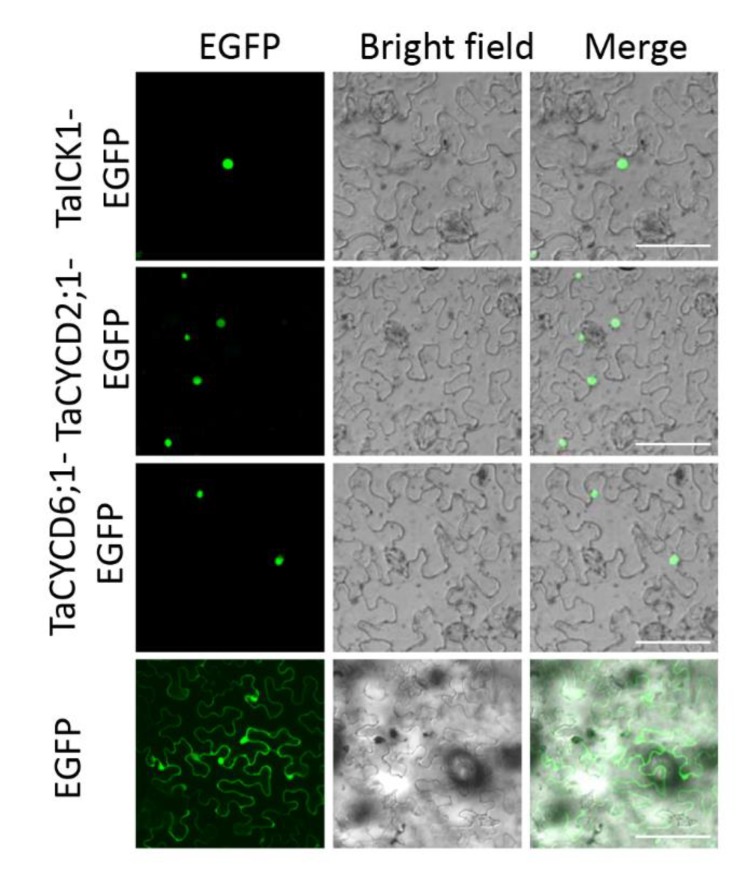
Subcellular localization of TaICK1, TaCYCD2;1, and TaCYCD6;1 in *N. benthamiana* cells. The fluorescence (EGFP), bright field, and merged images were obtained using a confocal microscope. The scale bars are 50 μm.

To determine the intracellular distribution of protein TaICK1, the *TaICK1-EGFP* construct was transformed into the leaf of *Nicotiana benthamiana*. Signals of the EGFP protein alone, as a positive control, were distributed throughout the cell (Figure 7). By contrast, the green fluorescence of the TaICK1-EGFP fusion protein was only detected in the nucleus (Figure 7).

### 2.7. Prokaryotic Expression of TaICK1 in E. coli

The expected molecular weight of TaICK1 is 24 kDa based on the coding sequence. The fusion protein TF-TaICK1 was expressed in the *E. coli* strain BL21 (DE3). As shown in Figure 3A, the migration of fusion protein corresponded with a molecular weight of about 75 kDa according to SDS-PAGE. Hence, the objective protein was approximately 27 kDa after subtracting 48 kDa corresponding to the fused trigger factor protein tag. This is approximate with the prediction based on the coding sequence.

### 2.8. *TaICK1* Interacts with *TaCYCD2;1* and TaCYCD6;1

**Figure 8:**
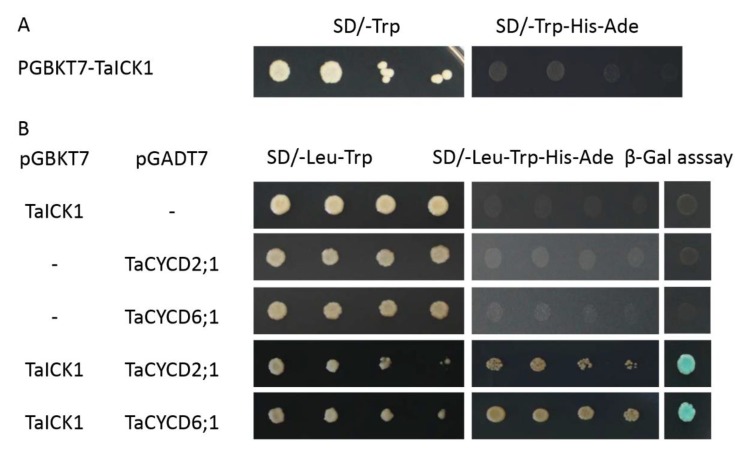
The interaction of TaICK1 with CYCD2;1 and CYCD6;1 in a yeast two-hybrid assay. (**A**) Transcriptional activation analysis of the TaICK1 in yeast. (**B**) The interaction of TaICK1 with TaCYCD2;1 and TaCYCD6;1 in the yeast two-hybrid assays.

The pGBKT7-TaICK1 plasmid failed to autonomously activate reporter genes in the yeast strain Y2H Gold (Figure 8A). Hence, this plasmid could be used to screen the yeast library. Ultimately, a total of 24 individual yeast clones were obtained, in which AF512432.1 (NCBI) and AK450777.1 (NCBI) were identified and annotated as *TaCYCD2;1* and *TaCYCD6;1*, respectively. *TaCYCD2;1* and *TaCYCD6;1,* which have been identified as being involved in the regulation of the cell cycle, were selected for further analysis. In addition, both the TaCYCD2;1-EGFP and TaCYCD6;1-EGFP fusion proteins were localized to the nucleus in the epidermis leaf cells of *Nicotiana benthamiana* (Figure 7). The respective protein molecular weights of TaCYCD2;1 and TaCYCD6;1 were approximately 38 and 33 kDa after subtracting the 48 kDa trigger factor protein tag (Figure 3B,C).

**Figure 9:**
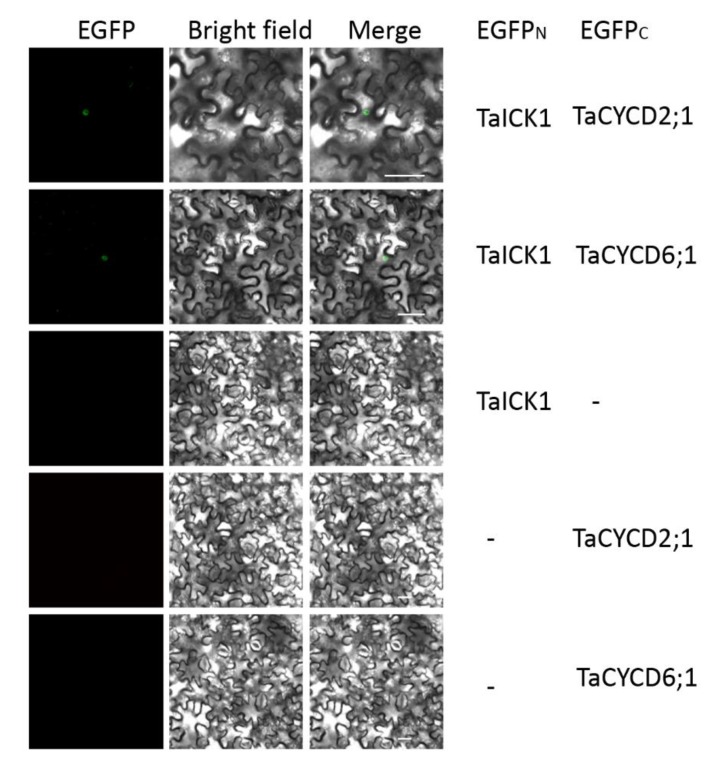
The interaction of TaICK1 with CYCD2;1 and CYCD6;1 in the BiFC system. Fluorescence (EGFP), bright field, and merged images were obtained using a confocal microscope. The scale bars are 50 µm.

The interactions between TaICK1 and TaCYCD2;1 and TaCYCD6;1 were confirmed in yeast (Figure 8B). The interactions between TaICK1 and CYCD2;1 and TaCYCD6;1 were further validated using a BiFC assay (Figure 9). pCAMBIA1302-35S*::*TaICK1-EGFP_N1–155_ and pCAMBIA1302-35S*::*TaCYCD2;1 -EGFP_C156–237_ or pCAMBIA1302-35S*::*TaCYCD6;1-EGFP_C156–237_ were co-expressed in *Nicotiana benthamiana* leaf cells. These results not only demonstrate that TaICK1 interacted with TaCYCD2;1 and TaCYCD6;1 but also suggest that the protein complexes of TaICK1 and TaCYCD2;1, as well as that of TaCYCD6;1, may act functionally in the nucleus.

### 2.9. The Expression Patterns of CYCD2;1 and CYCD6;1 in Wheat and Transgenic Rice

The expression levels of *CYCD2;1* and *CYCD6;1* were analyzed in the wheat and transgenic rice plants. As shown in Figure 5C, the expression level of *TaCYCD2;1* was lower in XN1376-CIMS than that in XN1376, especially at the Eun and Lun stages. In total, the trend of expression increased initially and then decreased from the Eun to Tn stage (Figure 5C). The expression of *TaCYCD6;1* was similar to that of *TaCYCD2;1* (Figure 5D). Furthermore, the expression levels of O*sCYCD2;1* and *OsCYCD6;1* were measured in transgenic and wild-type rice plants. The expression of *OsCYCD2;1* was higher in transgenic rice plants and wild-type plants at the Eun and Lun stages (Figure 6C), as was *OsCYCD6;1* (Figure 6D). Notably, in rice plants overexpressing *TaICK1,* the expression of *OsCYCD2;1* and *OsCYCD6;1* was significantly lower than that in wild-type plants (Figure 6C,D).

## 3. Discussion

CHA SQ-1 can effectively induce male sterility in most wheat varieties, reaching rates of up to 95%. Meanwhile, the seed setting rate of artificial saturation pollination can reach 98% [15]. This result shows the great potential of utilizing heterosis. However, a small amount of self-fertility will affect the purity of hybrid F1 seeds to some extent. Hence, it is urgently required to ameliorate the male sterility induced by SQ-1. Thereafter, we could improve the induction efficiency of male sterility to reduce this self-fertility.Therefore, several studies have investigated the molecular mechanism behind the sterility induced by SQ-1, including characterization of its physiological and biochemical metabolism characteristics [48,49] as well as the genes related to pollen grain development [19,24,50] and proteome [16,22,23,51] and epigenetic [52,53,54,55] profiles. However, the molecular mechanism underlying SQ-1-induced male sterility remains unclear.

In our study, we confirmed that TaICK1 is a potential contributor to wheat male sterility induced by SQ-1. A previous study indicated that ICK proteins are inhibitors of CDK proteins and can block CDK activity by binding, generating CDK–Cycd protein complexes [56]. Some studies have indicated that ICK1 expression level can be induced by ABA [37] or salt [57]. Interestingly, the expression level of *TaICK1* in XN1376-CIMS was significantly higher than that in the control plant XN1376 during the Eun, Lun, and Tn stages (Figure 5B). Especially at the Eun and Lun stages, the expression level of XN1376-CIMS was 2.8 and 1.7 times that of XN1376. These results report, for the first time, that CHA SQ-1 may be able to increase the expression of *TaICK1*. Moreover, as shown in Figure 1J, the pollen grains of XN1376-CIMS were only lightly stained while the pollen grains of XN1376 were deeply stained (Figure 1I). This is consistent with the results of the *OsiICK6* overexpression, which showed that the normal pollen grain ratio was only 22% in transgenic plants, which is lower than that in control plants [58]. Similar results were also observed in the *Arabidopsis ICK1* overexpression lines [59] and for overexpression of *Orysa;KRP1* in rice [36]. Together, these results suggest that the *ICK1* gene may act as a key potential contributor to male sterile formation. Meanwhile, previous studies have indicated that the expression levels of *ICKs* are crucially related to plant development. For example, increased *ICK1* expression resulted in reduced CDK activity and also a reduction in the overall number of cells in transgenic *Arabidopsis ICK1* plants [40]. A similar phenotype was observed in the transgenic *Arabidopsis* plants overexpressing *KRP2* (the leaves of mature transgenic plants are serrated with enlarged cells) [41]. Similar results were also obtained in our study. The development of *TaICK1* transgenic plants showed a difference compared with control rice plants. For example, the seed setting rate of *TaICK1^OE^*^-1^ decreased by 67% compared to the control plants (Table 1). This result is similar to the experiment showing that the transgenic lines overexpressing *OsiICK6* have a seed setting rate of only 27.7% on average [58]. Furthermore, the heading period was markedly delayed in *TaICK1* transgenic lines (Figure 2N). To the best of our knowledge, this is the first time that *ICK* genes have been implicated in the regulation of the heading period. Taken together, these results demonstrate that an excess of *ICKs* greatly impairs the seed setting rate [31,60]. Hence, these results also show that a higher level of *TaICK1* in XN1376-CIMS is closely related to male sterility.

Generally, cell division in a plant is controlled by CDK–cyclin protein complexes [56]. In the current study, the expression patterns of *TaICK1* in XN1376-CIMS after spraying with SQ-1 suggest that *TaICK1* might serve a key role in the process of SQ-1-induced physiological male sterility. Thus, it is necessary to determine whether TaICK1 can interact with the cyclins in wheat. Therefore, a yeast two-hybrid system was used to identify the possible interacting proteins by screening the yeast libraries. Interestingly, *TaCYCD2;1* and *TaCYCD6;1* were identified from common wheat by using the protein TaICK1 as the bait. It is known that D-type cyclins (Cycds) control the G1/S transition of the mitotic cell cycle in mammals and plants [61], as well as cell growth [62]. *CYCD2;1* increases cell formation in leaf [63] and accelerates the rate of leaf initiation [64], suggesting that *CYCD2;1* exerts a positive function on cell development. Furthermore, the increased expression of *CYCD* can suppress the negative effects of excessive ICK/KRP levels on plant growth [39,47]. In XN1376-CIMS, the expression levels of *TaCYCD2;1* and *TaCYCD6;1* were significantly lower than in XN1376 (Figure 5C,D). One possible explanation is that SQ-1 increased the expression level of *TaICK1* and also reduced the *TaCYCD2;1* and *TaCYCD6;1* expression levels in XN1376-CIMS. However, as shown in Figure 6C,D, the expression levels of *OsCYCD2;1* and *OsCYCD6;1* were also lower in *TaICK1* transgenic rice plants than in control plants. This result demonstrates that reduced *TaCYCD2;1* and *TaCYCD6;1* expression is not related to SQ-1.

In total, excess *TaICK1* impaired the sterility of anthers in XN1376-CIMS. In addition, *TaICK1* might suppress the expression level of *TaCYCD2;1* and *TaCYCD6;1* in the anthers of XN1376-CIMS, which might then impair the fertility and seed setting rate. This study offers new insights into SQ-1-related sterility in common wheat. However, more studies should be carried out to determine, in detail, the molecular mechanism underlying wheat sterility.

## 4. Materials and Methods

### 4.1. Plant Materials and Growth Conditions

The wheat cultivar XN1376 was planted in the experimental field of Northwest A&F University, Yangling, Shaanxi, China (108°E, 34°15′N) from 2015 to 2017 (as usual). Trenching was manual and planting was single. The plants were evenly distributed using this approach. SQ-1, a novel CHA, was readily dissolvable in water [49]. A total of 5.0 kg/ha SQ-1 was sprayed on leaves of wheat XN1376 at the 8.5 stage of the Feekes scale to produce male sterile XN1376-CIMS lines [48]. Meanwhile, XN1376 was treated with water to produce control lines. Cytological characteristics were investigated at the different microsporogenesis phases of male sterility and male fertility development. Fresh anthers from four different developmental stages (e.g., Eun, the early uninucleate stage; Lun, the late uninucleate stage; Bn, the binucleate stage; and Tn, the trinucleate stage) were collected and stored at −80 °C.

### 4.2. Phenotype Characterization of Wheat Anther Development

Photographs of the fresh anthers at each sequential stage were obtained from XN1376 and XN1376-CIMS with a Nikon E995 digital camera (Nikon, Tokyo, Japan) fixed firmly to a Motic K400 dissecting microscope (Preiser Scientific, Louisville, KY, United States). Pollen grains were also analyzed via 1% iodine–potassium iodide (1% KI–I_2_) staining to determine the viability of the mature pollen, as previously described [65]. Scanning electron microscopy was used to characterize the surface characteristics of the anthers. Before the experiment, fresh anthers and pollen grains were fixed in 4% glutaraldehyde and then treated with an alcohol gradient, dried, and broken in sequence. Finally, the anthers and pollen grains were mounted on a stub with colloidal silver and photographed using a JSM-6360LV scanning electron microscope (JEOL, Tokyo, Japan) [66].

### 4.3. RNA Extraction and qRT-PCR Analysis

Total RNA was extracted from the anthers of four developmental stages (i.e., Eun, Lun, Bn, and Tn) using TRIzol reagent (Takara Bio, Tokyo, Japan). The integrity of the RNA was assessed using electrophoresis on a 1.5% agarose gel. Meanwhile, the concentration and purity of the RNA was further determined using NanoDrop (Thermo Scientific, Wilmington, DE, USA). A PrimeScript^TM^ RT reagent Kit with a gDNA Eraser (Takara Bio, Tokyo, Japan) was used to synthesize the first-strand cDNA. 2× RealStar Green Power Mixture (GenStar BioSolutions Co., Ltd., China) was used to perform qRT-PCR. The reaction mixture was used according to the manufacturer’s protocols. The data were collected using a QuantStudio^TM^ Real-Time PCR System (Thermo Fisher, Waltham, MA, USA Applied Biosystems, USA) under the following PCR program: 95 °C for 10 min, followed by 40 cycles at 95 °C for 15 s, 60 °C for 30 s, and 72 °C for 30 s. All experiments were carried out with three technical replicates and three independent biological replicates. The relative expression levels of the genes were computed using the 2^−ΔΔCt^ method, as described by Livak and Schmittgen [67].

### 4.4. Primer Design

The gene *TaICK1* was identified from leaf cDNA of XN1376 based on the nucleotide sequence of the *ICK1* in *Aegilops tauschii* (NCBI reference sequence: XM_020298214.1). The primers used in this study were designed using Oligo7 software. The forward primer sequence was 5′-GCGAAGATGAGGAAGCAG-3′, and the reverse primer sequence was 5′-CATCATGCTCTGCTCACACGG-3′. Gene-specific primers for the genes *TaCYCD2;1* (GenBank AF512432.1) and *TaCYCD6;1* (GenBank XM_020345750.1) were also designed (Supplementary Table S1). The specific primers used for qRT-PCR are listed in Supplementary Table S2.

### 4.5. Plasmid Constructs

Plasmid pCAMBIA1301-35S*::TaICK1*, containing a hygromycin selection marker, was constructed to generate transgenic rice plants. A cauliflower mosaic virus 35S (35S) promoter was used to drive the expression of *TaICK1*. The pGBKT7-*TaICK1* construct was used as the bait to screen the yeast cDNA library of the wheat anthers. In order to confirm the interaction of TaICK1 with TaCYCD2;1 or TaCYCD6;1, the coding sequences of *TaCYCD2;1* and *TaCYCD6;1* were cloned into pGADT7 as the prey. To generate the subcellular localization vector, the full-length coding sequence of gene *TaICK1* (without the termination codon) was cloned into the expression vector backbone pCAMBIA1302 under the control of the 35S promoter to create a pCAMBIA1302-2×35S*::TaICK1-EGFP* construct to express the TaICK1-EGFP fusion protein. Likewise, the pCAMBIA1302-2×35S*::TaCYCD2;1-EGFP* and pCAMBIA1302-2×35S*::TaCYCD6;1-EGFP* constructs were also created. For BiFC vector construction, the full-length coding sequence of *TaICK1* was ligated with DNA of EGFP_N1–155_ to produce the pCAMBIA1302-TaICK1-EGFP_N1–155_ construct for fusion protein expression driven by the 35S promoter. The pCAMBIA1302-TaCYCD2;1-EGFP_C156–237_ and pCAMBIA1302-TaCYCD6;1-EGFP_C156–237_ constructs were similarly generated for fusion protein expression. The prokaryotic expression vector pCold-TF (TaKaRa, Japan) was used to express the proteins TaICK1, TaCYCD2;1, and TaCYCD6;1, which generates a fusion protein with an extra 48kD trigger factor protein tag. The details of primers used for vector construction are given in Supplementary Table S1.

### 4.6. Generation of the TaICK1 Overexpression Rice Plants

To generate transgenic plants, the plasmid pCAMBIA1301-35S*::TaICK1* was introduced into *Agrobacterium tumefaciens* strain EHA105 using electroporation [68], and positive transformants were identified using PCR assay. Nipponbare rice plants were transformed as described [69]. The transgenic seedlings were initially identified using PCR with the primers described in Supplemental Table S2. The transgenic lines were then further screened on an identification buffer containing 50 mg L^−1^ of hygromycin and 0.5 mg L^−1^ 6-BA [70]. The transgenic rice leaves were able to remain green in this buffer, while the leaves from the non-transgenic rice showed a chlorotic phenotype. The T_2_ plants derived from at least three independent T_1_ transgenic plants were used to characterize the phenotype and assay gene expression using three independent biological replicates.

### 4.7. Yeast Two-Hybrid Assay

The yeast cDNA library of the anther tissue was introduced into the yeast strain Y187. The analyses were performed according the manufacturer’s instructions (Clontech, USA). The transcription activation activity of the bait protein was analyzed through the yeast growth status and an α-galactosidase activity assay on medium SD (−Trp/−Ade/−His) plates. For yeast cDNA library screening, Y2H colonies containing the pGBKT7-TaICK1 vector were mated with yeast strain Y187 (cDNA library of Anthers tissue), according to the instructions of the Two-Hybrid System 3 (Clontech, USA). Then, the mating type was selected on a high-stringency medium SD (−Ade/−His/−Leu/−Trp). Finally, after cloning the full length CDS of the prey gene, the prey genes were reconstructed into pGADT7. To further verify the protein interactions, the pGBKT7-bait vector and pGADT7-prey vector were co-transformed into Y2H Gold yeast cells (yeast strain Y2H, Clontech, USA), and the positive clones were selected on the SD medium (−Leu/−Trp). Positive yeast clones were picked and spread on the SD medium (−Ade/−His/−Leu/−Trp/X-α-Gal) to assay for protein interactions.

### 4.8. Subcellular Localization and BiFC Assay

Subcellular localization vectors were transformed into *Agrobacterium tumefaciens* strain GV3101, which was cultured in an LB medium with appropriate antibiotics (50 mg L^−1^ Kan, 25 mg L^−1^ gentamycin, and 25 mg L^−1^ rifampicin) under shaking at 200 rpm at 28 °C for about 36 h. *Agrobacteria tumefaciens* was then centrifuged at 5000*g* for 5 min to collect the bacteria when the OD_600_ reached 0.6. The cells were resuspended in resuspension buffer (containing 10 mM MES, 10 mM MgCl_2_, and 200 mM acetosyringone) and incubated for 2 h at room temperature. The suspension of *Agrobacteria tumefaciens* was then used to infiltrate 4- to 6-week-old *Nicotiana benthamiana* leaves as previously described [71,72]. Similarly, for the BiFC assay, pairs of EGFP_N_ and EGFP_C_ fusion proteins were transiently co-expressed in the leaves of *N. benthamiana*. Infiltrated leaves were observed using a laser confocal scanning microscope Olympus IX83 confocal microscope (Olympus, Tokyo, Japan). The excitation and detection wavelengths for EGFP were 514 and 527 nm, respectively.

### 4.9. Recombinant Protein Expression in E. coli

Plasmids pCold-TF-TaICK1, pCold-TF-TaCYCD2;1, and pCold-TF-TaCYCD6;1 were introduced into the *E. coli* BL21 (DE3) strain (TransGen Biotech, China) via the heat shock method, as described by the manufacturer. Colonies of verified transformants were incubated in LB [73] medium containing 50 mg L^−1^ carbenicillin at 37 °C for 8–12 h until the optical density OD_600_ reached 0.6–0.8. IPTG (Isopropyl β-D-Thiogalactoside) was then added into the medium at a final concentration of 0.5 mM, followed by incubation at 16 °C overnight with shaking at 100 rpm. The control culture was treated using the same protocol without IPTG. After incubation, the crude cell extracts were prepared as described [74]. Then, the crude proteins were analyzed using SDS-PAGE and dyed with Coomassie brilliant blue R-250 using the protocol of Sambrook and Russell [74].

## 5. Conclusions

In this study, the wheat *TaICK1* gene was isolated and characterized. Moreover, the chemical hybridization agent SQ-1 was shown to induce male sterility in common wheat, as well as a higher expression level of *TaICK1* in the anthers of sterile wheat lines. More importantly, the overexpression of TaICK1 suppressed the expression of *TaCYCD2;1* and *TaCYCD6;1* and reduced the fertility seed setting rates. These results provide new insights into the molecular mechanism of wheat male sterility induced by the chemical hybridization agent SQ-1.

## Figures and Tables

**Table 1:** The seed-setting rate of the TaICK1 over-expressing plants and wild-type plants.

Plant	OE-1	OE-3	OE-5	OE-7	OE-8	OE-10	OE-15	WT
T_1_	19.7%	58.6%	58.7%	63.5%	38.1%	57.4%	29.6%	94.1%
T_2_	33.4%	58.6%	66.2%	46.7%	47.3%	30.2%	68.9%	95.1%

OE: overexpression plants; the number following OE is the code of the independent overexpression plants.

## Supplementary Materials

Supplementary materials can be found at https://www.mdpi.com/1422-0067/21/7/2468/s1. **Table S1.** Primers for gene-specific and plasmid construction in this study; **Table S2.** Primers used for quantitative real-time RT-PCR analysis.
