# Supplementary material for: Cyclin-Dependent Kinase Inhibitor Gene TaICK1 acts as a Potential Contributor to Wheat Male Sterility induced by a Chemical Hybridizing Agent
Source: Int J Mol Sci. 2020 Apr 2;21(7):2468. doi: 10.3390/ijms21072468 (PMC7177297; doi:10.3390/ijms21072468)
Supplement: Supplementary file 1 [file ijms-21-02468-s001.pdf]

Up-regulated cyclin-dependent kinase inhibitor gene *TaICK1* induced by chemical hybridization agent in wheat anthers acts as a potrntial contributor to male sterity

Lili Zhang[1,2,3,4†], Chaojie Wang[1†], Yongang Yu[5†], Yamin Zhang[1], Yulong Song[1], Zheng Li[1], Shuping Wang[6], Yanfang Zhang[1], Xiaofeng Guo[1], Dan Liu[1], Ziliang Li[1], Shoucai Ma[1], Jinjuan Zheng[1] and Gaisheng Zhang[1,2,3,4*]

[1]College of Agronomy, Northwest A & F University, National Yangling Agricultural Biotechnology & Breeding Center, Yangling, 712100, Shaanxi, P.R.China.
[2]Yangling Branch of State Wheat Improvement Centre, Yangling, 712100, Shaanxi, P.R.China.
[3]Wheat Breeding Engineering Research Center, Ministry of Education, Yangling, 712100, Shaanxi, P.R.China.
[4]Key Laboratory of Crop Heterosis of Shaanxi Province, Yangling, 712100, Shaanxi, P.R.China.
[5]Department of Life Sciences, Henan Institute of Science and Technology, Xinxiang, 453000, Henan, P.R.China.
[6]Hubei Key Laboratory of Waterlogging Disaster and Agricultural Use of Wetland, College of Agronomy, Yangtze University, Jingzhou, 434000, Hubei, P.R.China.

Supplemental Information

Table S1. Primers for gene-specific and plasmid construction in this study

Primer	Sequence (5′-3′)
	**Primers for gene-specific**
TaCYCD2;1-F	ATGGTTCCGTCCGGGTAC
TaCYCD2;1-R	TCATCTGCCAATTCTCCTCCT
TaCYC6;1-F	ATGGACATGCCGGGGAAGG
TaCYC6;1-R	CTACCCCCACTGGGACTGGGAAG
	**Primers for plant expression plasmids**
TaICK1-1301-F	CCATTTACGAACGATAGCATGAGGAAGCAGCAGCAGGCG
TaICK1-1301-R	CGAGATCTGAGTCCGGACTCACACGGTGGCCACCGGGGTC
	**Primers for prokaryotic expression plasmids**
TaICK1-pCold-TF-F	ACCCTCGAGGGATCCGAATTCATGAGGAAGCAGCAGCAGGCG
TaICK1-pCold-TF-R	CTATCTAGACTGCAGGTCGACTCACACGGTGGCCACCGGGGTC
TaCYCD2;1-pCold-TF-F	ACCCTCGAGGGATCCGAATTCATGGTTCCGTCCGGGTAC
TaCYCD2;1-pCold-TF-R	CTATCTAGACTGCAGGTCGACTCATCTGCCAATTCTCCTCCT
TaCYC6;1-pCold-TF-F	ACCCTCGAGGGATCCGAATTCATGGACATGCCGGGGAAGG
TaCYC6;1-pCold-TF-R	CTATCTAGACTGCAGGTCGACCTACCCCCACTGGGACTGGGAAG
	**Primers for subcellular localization plasmids**
TaICK1-1302EGFP-F	ACGGGGGACTCTTGACCATGGAGATGAGGAAGCAGCAGCAGGC
TaICK1-1302EGFP-R	AAGTTCTTCTCCTTTACTAGTCACGGTGGCCACCGGGGTC
TaCYCD2;1-1302EGFP-F	ATTTACGAACGATAGCCATGGACATGGTTCCGTCCGGGTAC

TaCYCD2;1-1302EGFP-R	AGATCTGAGTCCGGACCATGGTCTGCCAATTCTCCTCCT
TaCYC6;1-1302EGFP-F	ATTTACGAACGATAGCCATGGACATGGACATGCCGGGGAAGG
TaCYC6;1-1302EGFP-R	AGATCTGAGTCCGGACCATGGCCCCCACTGGGACTGGGAAG
**Y2H plasmids primers**	
TaICK1-BD-F	CATGGAGGCCGAATTCATGAGGAAGCAGCAGCAGGCG
TaICK1-BD-R	ATGCGGCCGCTGCAGGTCGACGCGTCTCTTTTCTCTCCC
TaCYCD2;1-AD-F	TGGAGGCCAGTGAATTCATGGTTCCGTCCGGGTAC
TaCYCD2;1-AD-R	TCGAGCTCGATGGATCCTCTGCCAATTCTCCTCCT
TaCYC6;1-AD-F	TGGAGGCCAGTGAATTCATGGACATGCCGGGGAAGG
TaCYC6;1-AD-R	TCGAGCTCGATGGATCCCCCCCACTGGGACTGGGAAG
**BiFC plasmids primers**	
TaICK1-BIFC-F	CACGCTGAAATCACCAGGTGCGAAGATGAGGAAGCAGCA
TaICK1-BIFC-R	CTCGCCCTTGCTCACCATCTCTGCTCTCACGGTGGCCAC
TaCYCD2;1-BIFC-F	CACGCTGAAATCACCAGGATGGTTCCGTCCGGGTAC
TaCYCD2;1-BIFC-R	CTTCTGCTTGTCGGCCATTCTGCCAATTCTCCTCCT
TaCYC6;1-BIFC-F	CACGCTGAAATCACCAGGATGGACATGCCGGGGAAGGAC
TaCYC6;1-BIFC-R	CTTCTGCTTGTCGGCCATCCCCCACTGGGACTGGGAAG

Table S2. Primers used for quantitative real-time RT-PCR analysis

Primer	Sequence (5′-3′)
TaICK1-RT-F	CGTCAGCGACTCTGAGTGCG
TaICK1-RT-R	TCCTGCGTTTGTGCTTCTGC
TaCYCD2;1-RT-F	CTCCATGCTCGCATTCTC
TaCYCD2;1-RT-R	CTTAACACTCGCTCCTTG
TaCYC6;1-RT-F	GGACGAGGAGTTCATGTTCGA
TaCYC6;1-RT-R	GAAGCACGCCGAGAGGAAGAA
OsICK1-RT-F	CTCCATCAAGCTTTCTCCCCG
OsICK1-RT-R	TTGCTGTGCTGAGGCTGTTG
OsCYCD2;1-RT-F	GAGAGGGTATTACGGTGCTATG
OsCYCD2;1-RT-R	TGGAATGGAGAAAACGGAGC
OsCYC6;1-RT-F	GGGCGAGGAGTTCATGTTCGA
OsCYC6;1-RT-R	GAAGCACGCGGAGAGAAAGAA
TaActin-F	TGTTGTTCTCAGTGGAGGTTCT
TaActin-R	CTGTATTTCCTTTCAGGTGGTG
OsActin-F	AACTGGGATGATATGGAGAA
OsActin-R	CCTCCAATCCAGACACTGTA
